# Amelioration of Alcoholic Hepatic Steatosis in a Rat Model via Consumption of Poly-γ-Glutamic Acid-Enriched Fermented *Protaetia brevitarsis* Larvae Using *Bacillus subtilis*

**DOI:** 10.3390/foods14050861

**Published:** 2025-03-03

**Authors:** So-Yeon Sim, Hyun-Dong Cho, Sae-Byuk Lee

**Affiliations:** 1 School of Food Science and Biotechnology, Kyungpook National University, 80 Daehak-ro, Daegu 41566, Republic of Korea; salley4565@knu.ac.kr; 2 Department of Food and Nutrition, Sunchon National University, Sunchon 57922, Republic of Korea; hdcho@scnu.ac.kr; 3 Institute of Fermentation Biotechnology, Kyungpook National University, 80 Daehak-ro, Daegu 41566, Republic of Korea

**Keywords:** alcoholic hepatic steatosis, *Bacillus subtilis*, fermentation, lipid metabolism modulation, poly-γ-glutamic acid, *Protaetia brevitarsis* larvae

## Abstract

Alcoholic hepatic steatosis (AHS) is a common early-stage symptom of liver disease caused by alcohol consumption. Accordingly, several aspects of AHS have been studied as potential preventive and therapeutic targets. In this study, a novel strategy was employed to inhibit fatty liver accumulation and counteract AHS through the consumption of microorganism-fermented *Protaetia brevitarsis* larvae (FPBs). By using an AHS rat model, we assessed the efficacy of FPB by examining the lipid profile of liver/serum and liver function tests to evaluate lipid metabolism modulation. After FPB administration, the lipid profile—including high-density lipoprotein, total cholesterol, and total triglycerides—and histopathological characteristics exhibited improvement in the animal model. Interestingly, AHS amelioration via FPBs administration was potentially associated with poly-γ-glutamic acid (PγG), which is produced by *Bacillus* species during fermentation. These findings support the formulation of novel natural remedies for AHS through non-clinical animal studies, suggesting that PγG-enriched FPBs are a potentially valuable ingredient for functional foods, providing an ameliorative effect on AHS.

## 1. Introduction

Alcoholic hepatic steatosis (AHS) is a common liver disease caused by the accumulation of triglycerides due to decreased oxidation and increased synthesis of fatty acids in the liver [1]. Furthermore, elevated levels of reactive oxygen species can cause chronic liver disease by damaging liver tissue [2]. Alcohol, a major factor in causing alcoholic hepatic steatosis, is primarily metabolized in the liver [3]. Alcohol consumption brings about toxins, such as acetaldehyde, during its breakdown in the body, leading to various disorders in hepatic cells, including hangovers. With chronic drinking, these conditions often result in serious alcoholic liver diseases, such as AHS [4]. In our current complex social structure, an increasing number of people who suffer from poor health because of frequent alcohol consumption have recently sought remedies, such as liver function-improving agents and health-promoting foods. Furthermore, several researchers have attempted to identify natural hepatoprotective substances that can protect the liver against alcohol-induced toxicity [5].

Edible insects are widely consumed as traditional foods in various countries, and their popularity as an alternative food source is steadily growing [6]. They are reported to possess a higher nutritional value than conventional meat, including elevated levels of protein, essential amino acids, minerals, and unsaturated fatty acids [7]. Among edible insects, *Protaetia brevitarsis* larvae (PbsLs) have been approved as a safe food ingredient by the Korea Food and Drug Administration after thorough safety evaluations. They demonstrate antioxidant, anti-coagulant, and anti-inflammatory activities and serve as a rich source of protein [8]. Although PbsLs’ potential to enhance the liver function has long been recognized, scientific evidence supporting its ability to ameliorate alcoholic fatty liver disease is yet to be documented.

Fermentation is a traditional food processing technique that improves food quality by enhancing the synthesis of new physiologically active components, such as antioxidants, and reducing food toxicity through the metabolic systems of microorganisms such as filamentous fungi, yeasts, lactic acid bacteria, and bacilli [8]. *Bacillus* species, which are commonly used in fermentation, exist in foods like cheonggukjang and natto, and they generate various active components during proliferation [9]. Growing *Bacillus* species cells frequently produce poly-γ-glutamic acid (PγG), a natural biopolymer comprising D- and L-glutamic acid in different ratios [10]. In a previous study, “high-fat diet-containing PγG” group subjects experienced decreased levels of total cholesterol, low-density lipoprotein (LDL)-cholesterol, and triglycerides along with increased in high-density lipoprotein (HDL)-cholesterol levels compared with “high-fat diet-only” group subjects [11]. These findings suggest a positive effect of PγG on lipid metabolism, increasing interest in its potential use.

Given the established positive effects of PγG on lipid metabolism, this study aims to assess the synergistic abilities of *Bacillus subtilis*, a producer of PγG, and PbsLs for the improvement of AHS. We fermented PbsLs using *Bacillus subtilis* and applied it in an alcohol-induced fatty liver rat model to analyze its effects on their lipid metabolism functions. This study validates fermented PbsLs (FPBs) as a potential functional food ingredient for the treatment of AHS.

## 2. Materials and Methods

### 2.1. Fermentation Conditions

In this study, PbsL powder was purchased from a native village (Namyangju, Republic of Korea) in June 2017 and used as the experimental material. *Bacillus subtilis* KACC 91157 (Dong-A University, Busan, Republic of Korea) was used as the fermentation strain. For the fermentation process, *B. subtilis* was cultured in a natural medium containing 5% (*w*/*w*) soybean. Microbial pellets (10%, *v*/*v*) were collected from the microbial medium via centrifugation, resuspended in sterilized distilled water, and used to inoculate the PbsL powder. Fermentation progressed in an incubator at 37 °C for 3 days, and sterilized distilled water was mixed with the fermenting powder to maintain the moisture content. After 3 days, the FPBs were dried at 50 °C for 5 h to yield the powder used for further experimentation.

### 2.2. PγG and Free Glutamic Acid Analyses

Total and free glutamic acids were analyzed using an L-8900 amino acid analyzer (Hitachi-Hitech, Tokyo, Japan) with an ion-exchange resin column (60 × 4.6 mm, sodium type). The detection wavelength was 570 nm, and the mobile phase comprised a lithium citrate buffer mixed with pyridine hydrochloric acid. The flow rate was maintained at 0.4 mL/min, with 10 µL of sample injected into the column. The column temperature was set at 57 °C, while the reactor temperature was maintained at 135 °C. The entire analytical procedure was completed within 33 min. The analysis results of total and free glutamic acid, as well as protein-bound and free amino acids, are presented in Tables S1 and S2. The PγG content (%) was calculated as PγG content (%) = [total glutamic acid content (%) × 0.88] − free glutamic acid content (%).

### 2.3. DPPH Radical Scavenging Assay

DPPH (2,2-diphenyl-2-picrylhydrazyl) radical scavenging activity was analyzed using a colorimetric assay [12]. Samples were added to 200 µL of a 0.2 mM DPPH solution, and the resulting mixture was reacted in the dark at 25 °C for 20 min. Absorbance (in optical density [OD]) was subsequently measured at 528 nm, using butylated hydroxytoluene (BHT) as a positive control. The percentage scavenging activity of DPPH was calculated as

Scavenging activity (%) = [(1 − Sample OD)/(Control OD)] × 100

### 2.4. Biological Properties

The flavonoid and total polyphenol content was measured using colorimetric assays. The sample extract was mixed with a 5% (*w*/*v*) NaNO_2_ and 10% AlCl_3_·6H_2_O solution and allowed to react for 30 min; for flavonoids [13], absorbance was measured at 510 nm using (+) catechin hydrate as a standard for the calibration curve. For total polyphenols [14], absorbance was measured at 760 nm using tannic acid as the standard. The flavonoid and total polyphenol contents were both expressed as mg% of the control.

### 2.5. Antithrombotic Activity

Fibrin plates were prepared by mixing 0.06% (*w*/*v*) fibrinogen with a 0.05 M sodium borate buffer (pH 7.8) and 40 units/mL thrombin. To form a fibrin clot, the plates were maintained at 25 °C for 15 min. Ten 10 µL of sample was then dropped onto a paper disc and incubated for 24 h at 37 °C. The activity of the fibrinolytic area was calculated as units/mL relative to the standard.

### 2.6. Cell Culture

Neutral lipid accumulation was determined using a human hepatocellular carcinoma (HepG2) cell line obtained from the Korean Cell Line Bank (Seoul, Republic of Korea). Cells were cultured in Dulbecco’s modified Eagle’s medium supplemented with 100 U/mL of an antibiotic-antimycotic solution (GIBCO^®^, Thermo Fisher Scientific, Waltham, MA, USA) and 10% fetal bovine serum. The cells were incubated at 37 °C in an incubator (Thermo Fisher Scientific) containing a 5% CO_2_ in a humidified atmosphere and then seeded onto a 96-well plate at a density of 5 × 10^4^ cells/well for 24 h. The cells were subsequently incubated at a 0.5% (*v*/*v*) sample concentration for 4 h. After incubation, the cell medium was replaced with a solution containing 5 mM ethyl alcohol to induce neutral lipid accumulation and incubated for 5 days.

### 2.7. Cellular Neutral Lipid Accumulation

To assess intracellular neutral lipid accumulation, Oil Red O staining was performed. The cultured HepG2 cells were treated with 4% (*v*/*v*) paraformaldehyde for 30 min. Subsequently, the cells were exposed to an Oil Red O staining solution and incubated in darkness for 1 h. Following the staining process, the cells were rinsed with phosphate-buffered saline to remove unbound Oil Red O stain and visualized at 200× magnification using an optical microscope (Leica Microsystems, Wetzlar, Germany). Images were captured for documentation. For quantitative analysis, isopropanol was added to 60% (*v*/*v*) in each well of a 96-well plate containing stained samples, and the optical density of the samples was determined at 540 nm using a microplate reader (Molecular Devices Inc., Sunnyvale, CA, USA).

### 2.8. Animals, Experimental Design, and Diet

Five-week-old, male, white Sprague–Dawley rats were purchased from Hyochang Science (Daegu, Republic of Korea). Rats at approximately 5 weeks old are transitioning their maturation phase, while rats at 6 weeks are considered fully mature. To minimize potential confounding factors such as dietary changes, aging, and diseases, we excluded older rats from this study. Therefore, rats at this stage, after passing the rapid growth phase of infancy, were chosen to induce chronic alcohol intoxication. Furthermore, we chose this animal model based on previous research that explored the ameliorative effects of fermented PbsLs on non-alcoholic fatty liver disease [15]. By using the same model, we aimed to maintain consistency in evaluating the physiological and metabolic effects of FPBs in the context of alcohol-induced liver disease. In this experiment, they were randomly classified according to their weight and raised in animal breeding rooms at a controlled temperature of 22 ± 2 °C and humidity of 50% and under a 12 h light/dark cycle (07:00–19:00/19:00–07:00). The rats were provided with a semi-purified diet for 1 week before the experiment. Their daily food intake was measured during the experiment, and weights were measured once a week. Our animal experiments followed the National Institute of Health guidelines, and this study was approved by Dong-A University’s Ethical Review Committee for Animal Experiments (DIACUC-17-23). The rats were categorized into normal (N), alcohol-only control (C), silymarin (PC), non-fermented PbsL (NPB), and FPB (FPB) treatment groups. Silymarin, which promotes liver regeneration, was used as a positive control for AHS mitigation. Apart from N, which received no alcohol, the other groups were fed experimental diets containing alcohol and specific additives depending on their treatment group: 100 mg/kg silymarin (PC), 400 mg/kg PbsL (NPB), 100 and 400 mg/kg FPBs (FPB100 and FPB400). To prevent acute alcohol toxicity, the intake concentration of alcohol was gradually increased from 10% in the first week to 20% in the second week; finally, the rats were fed the manufactured diet containing 30% alcohol for the final 4 weeks of the experiment. The compositions of the experimental diets are presented in Table 1 and Table 2. In Table 2, the term “normal diet” mentioned in this study refers to the diet composition presented in Table 1. All groups except for the N group (C, PC, NPB, FPB100, and FPB400) were subjected to alcohol-induced disease. The term “normal diet” was used to emphasize that the N group did not undergo disease induction. Instead, the N group was fed the baseline diet.

### 2.9. Dissection and the Analytical Procedure

All food was removed from the cages 12 h before dissection. For dissection, the rats were first anesthetized with diethyl ether, blood was withdrawn from the aorta, and then they were sacrificed. The tissues were extracted, cleaned with a 0.9% (*w*/*v*) saline solution, weighed, and then frozen. The blood was allowed to rest at room temperature for 30 min and subsequently centrifuged at 3000 rpm for 20 min. The serum that was collected via centrifugation was subjected to biochemical analysis to determine its biochemical properties; lipid metabolism; and total protein, globulin, and albumin levels, which were measured by the South-East Medi-Chem Institute (Busan, Republic of Korea) and Neodin BioVet. Laboratory (Seoul, Republic of Korea). Total lipid and free fatty acid concentrations were measured by Seoul Clinical Laboratories (Seoul, Republic of Korea).

### 2.10. Analysis of Alcohol-Metabolizing Enzymes

Liver tissues were homogenized in an ice-cold 9× potassium phosphate buffer (0.1 mM potassium phosphate, 1 mM sodium ethylenediaminetetraacetic acid, and 1 mM dithiothreitol, pH 7.4). After centrifuging the homogenate solution, the tissue residue and supernatant were separated. The mitochondrial fraction was acquired via centrifugation at 600× *g* for 20 min, 1000 rcf for 20 min, and 12,000 rpm for 10 min, in that order. Cytosol and microsomal fractions were extracted from the final supernatant for use as experimental material. The protein concentrations of the liver tissues were measured using the method described by Lowry et al. (1951) [16]. Bergmeyer’s method, which measures the rate of nicotinamide adenine dinucleotide-reduced form (NADH) generation at an absorbance of 340 nm, was slightly modified to measure alcohol dehydrogenase (ADH) activity in the liver tissues [17]. The aldehyde dehydrogenase (ALDH) activity of the liver tissues was measured based on the generation of NADH from NAD using the method of Koivula with modifications [18].

### 2.11. Serum Alcohol Concentration

Serum alcohol and acetaldehyde concentrations were determined using a commercial UV-test kit (R-Biopharm, Darmstadt, Germany). First, NADH formation was measured quantitatively by increasing the absorbance to 340 nm. Then, 3 mL of a phosphate buffer (pH 9.0) mixture was added to a solution containing an NAD tablet and 0.1 mL of serum, and the samples were allowed to react at room temperature for 3 min. To observe the changes in NADH concentration, 0.05 mL of ADH was added to the recovered solution to enable absorbance determination, and after 10 min of reaction at room temperature, absorbance at 340 nm was measured. To determine the acetaldehyde level, we added 3 mL of a phosphate buffer (pH 9.0) mixture to a solution containing an NAD tablet and 0.2 mL of serum and measured its absorbance at 340 nm. To observe the changes in NADH concentration, 0.05 mL of ALDH was added to the recovered solution. And after 5 min of reaction at room temperature, absorbance at 340 nm was measured. The standard concentrations of alcohol and acetaldehyde were calculated using the standard solutions provided by the supplier.

### 2.12. Liver Histopathological Study

After dissection, the liver tissues were washed with a cool physiological saline solution to remove the blood. Subsequently, a portion of the tissue was fixed in a 10% (*w*/*v*) formalin solution. After embedding the samples in paraffin, each was sliced into 3–4 μm-thick sections for staining with hematoxylin and eosin. Histopathological images were observed using an optical microscope (Olympus BX41; Olympus Co., Tokyo, Japan).

### 2.13. Statistical Analysis

Data generated from the animal experiments are presented as the mean ± the standard error (SE) of six replicates. Differences between treatments were assessed using one-way analyses of variance, with Duncan’s new multiple-range test used for pairwise mean comparisons. A *p*-value < 0.05 was accepted as indicating significant differences.

## 3. Results

### 3.1. PγG Content of FPBs at Different Fermentation Times

The PγG content of the FPB was measured each day during the 3-day fermentation period (Table 3). On the third day, the PγG content was highest, at 65.50 mg/g, which was 38% higher than that of the NPB group (47.17 mg/g).

### 3.2. Effect of Fermentation on the DPPH Radical Scavenging and Antithrombotic Activities of PbsLs

The antioxidant activity of PbsLs after fermentation using various microorganisms was evaluated (Table 4). The positive control (BHT) showed 87.9% activity. In comparison, non-fermented PbsLs exhibited 14.05% activity, and fermentation significantly increased the average antioxidant activity to 53.59%. To quantify antithrombotic activity, the diameter of the clear zone formed by thrombus dissolution was converted into units. Compared to the non-fermented PbsLs (2.88 units), fermentation using all bacterial strains significantly increased antithrombotic activity: *Lactobacillus plantarum* F3 and F5 yielded 39.20 units; *L. gaseri* yielded 42.00 units; and *Aspergillus kawachii* and *Saccharomyces cerevisiae* both yielded 33.80 units. The PbsLs fermented using *B. subtilis*, producing 64.80 units, exhibited the highest antithrombotic activity, representing a 22.5-fold increase compared to the N group.

### 3.3. Biological Properties of FPBs

**Figure 1:**
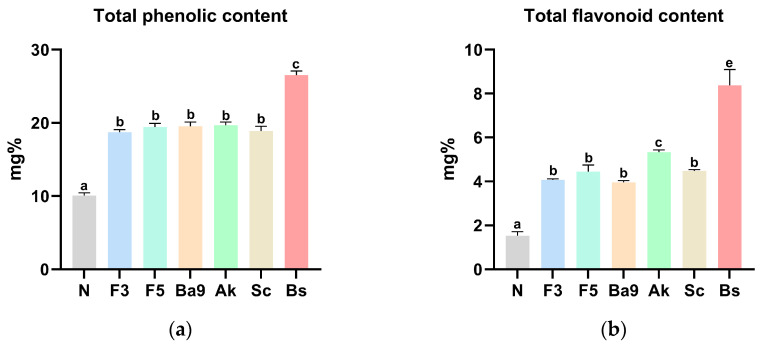
Biological properties of fermented *Protaetia brevitarsis* larvae (PbsLs) fermented for 3 d using different microbial species: total phenolic compound (**a**) and flavonoid contents (**b**). Values are presented as the mean ± standard error (*n* = 3). Means with the same letter are not significantly different (α = 0.05), as determined using one-way analyses of variance followed by Duncan’s new multiple-range tests. Abbreviations include N, non-fermented; F3, PbsLs fermented by *Lactobacillus plantarum* JBMI F3; F5, PbsLs fermented by *L. plantarum* JBMI F5; Ba9, PbsLs fermented by *L. gaseri* Ba9 F3; Ak, PbsLs fermented by *Aspergillus kawachii* KCCM 32819; Sc, PbsLs fermented by *Saccharomyces cerevisiae* KACC 93023; and Bs, PbsLs fermented by *Bacillus subtilis* KACC 91157.

Compared with that of the non-fermented PbsLs (10.07 mg%), fermentation using all bacterial strains except *B. subtilis* increased the average phenolic compound content by approximately 19.25%, with *B. subtilis* producing significantly higher total phenolic compounds (Figure 1a). The total flavonoid content displayed a similar trend to the polyphenol content, with *B. subtilis* producing an approximately 5.4-fold higher content (8.38 mg%) than that of the non-fermented PbsLs (1.53 mg%) (Figure 1b).

### 3.4. Effect of FPBs on the Inhibition of Neutral Lipid Accumulation in HepG2 Cells

In the alcohol-treated HepG2 cells, lipid droplets were stained using an Oil Red O reagent. Cells treated only with alcohol experienced the highest lipid accumulation rate, with FPB-treated cells demonstrating significantly lower rates (Table 5). Notably, the cells treated with 3-day-fermented PbsLs showed a marked reduction in neutral lipid accumulation, indicating a strong protective effect of the FPB.

### 3.5. Body Weight Gain, Relative Liver Weight, and Water Consumption of the Experimental Rats

The body weight gains and relative liver weights of the rats were measured as indicators of alcoholic hepatic toxicity (Table 6). The N-group rats exhibited the highest body weight, while C-group rats, which received only alcohol, had the lowest body weight. This is attributed to nutritional imbalances caused by alcohol consumption. Among the treatment groups, the PC and NPB groups both showed a recovery in body weight compared to the C group, but the FPB100 group demonstrated the most significant weight increase. In contrast, the FPB400 group did not exhibit significant changes in body weight compared to the C group. The C group exhibited the highest relative liver weight, 3.76%, which was significantly higher than those of the N, PC, and FPB400 groups. Additionally, despite having the highest body weight, FPB100-group rats had the lowest relative liver weight, 3.08%. Throughout the experimental period, water consumption was lower in all treatment groups compared with that of the N group, with no significant differences observed between the treatment groups.

### 3.6. Effect of FPBs on Serum Lipid Profiles

**Figure 2:**
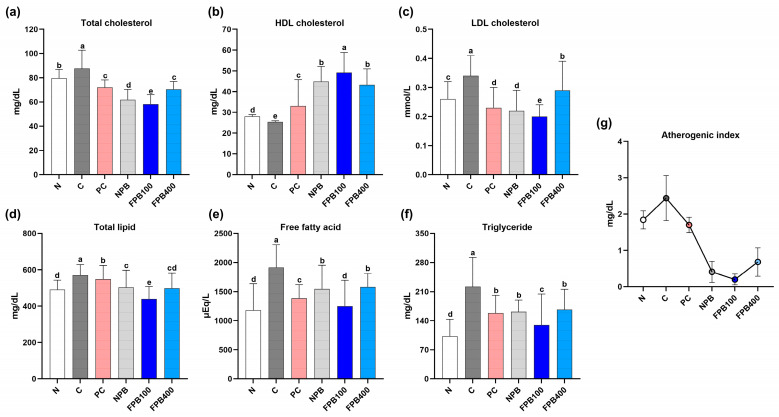
Effect of fermented *Protaetia brevitarsis* larvae (PbsLs) on the serum lipid profiles of alcohol-exposed rats: total cholesterol (**a**), HDL cholesterol (**b**), LDL cholesterol (**c**), total lipids (**d**), free fatty acids (**e**), triglycerides (**f**), and atherogenic index (**g**). Values are presented as the mean ± standard error (*n* = 6). Means with the same letter are not significantly different (α = 0.05), as determined using one-way analyses of variance followed by Duncan’s new multiple-range tests. Treatments include N, normal; C, control (alcohol only); PC, positive control (silymarin); NPB, non-fermented PbsLs combined with *Bacillus subtilis* KACC 91157; and FPB100 and FPB400, 100 and 400 mg/kg/d, respectively, of PbsLs fermented by *B. subtilis* for 3 d.

Figure 2a–g represents the serum lipid profile of the experimental rats, including total cholesterol, HDL cholesterol, LDL cholesterol, total lipid, free fatty acid, and triglyceride levels. The total cholesterol levels of the N and C groups were 79.64 ± 7.23 and 87.61 ± 15.12 mg/dL, respectively. The FPB treatments decreased cholesterol levels, with the FPB100 group having the lowest. The LDL cholesterol levels followed a similar trend: The levels in the N and C groups were 0.26 ± 0.06 and 0.34 ± 0.07 mg/dL, respectively. While the PC group showed a reduced LDL cholesterol level, the FPB100 treatment yielded the lowest (0.20 ± 0.04 mg/dL). Conversely, HDL cholesterol levels showed an opposite trend: The FPB groups exhibited higher levels than the N and C groups, with the FPB100 treatment producing the highest. Conversely, the FPB400 group exhibited slightly higher total cholesterol and LDL cholesterol levels compared to the FPB100 group, while HDL cholesterol levels were slightly lower than those in the FPB100 group. The atherogenic index (AI), calculated as AI = (total cholesterol − HDL-cholesterol)/HDL-cholesterol, followed a trend similar to those of total and LDL cholesterol.

The total lipid concentration of the C group (571.00 ± 56.86 mg/dL) exceeded that of the N group (491.38 ± 52.27 mg/dL) owing to the alcohol consumption, and the FPB100-group rats exhibited the lowest total lipid levels (440.00 ± 67.80 mg/dL). At the same time, the higher FPB-concentration group (FPB400) displayed a rapid increase in their total lipid concentrations. As illustrated in Figure 2e, free fatty acid levels showed a trend similar to total and LDL cholesterol, with the FPB100 group exhibiting the lowest levels. Triglyceride levels in C-group rats (221.93 ± 70.37 mg/dL) greatly exceeded those in the N-group rats (102.57 ± 40.79 mg/dL) (Figure 2f), and all treatments reduced triglyceride concentrations below that of the C group, with the FPB100-group rats showing the greatest reductions. Similarly, the FPB400 group exhibited slightly higher total cholesterol and LDL cholesterol levels compared to the FPB100 group, following the same trend observed in total lipid and free fatty acid levels.

### 3.7. Effect of FPBs on Liver Function Test Results

**Figure 3:**
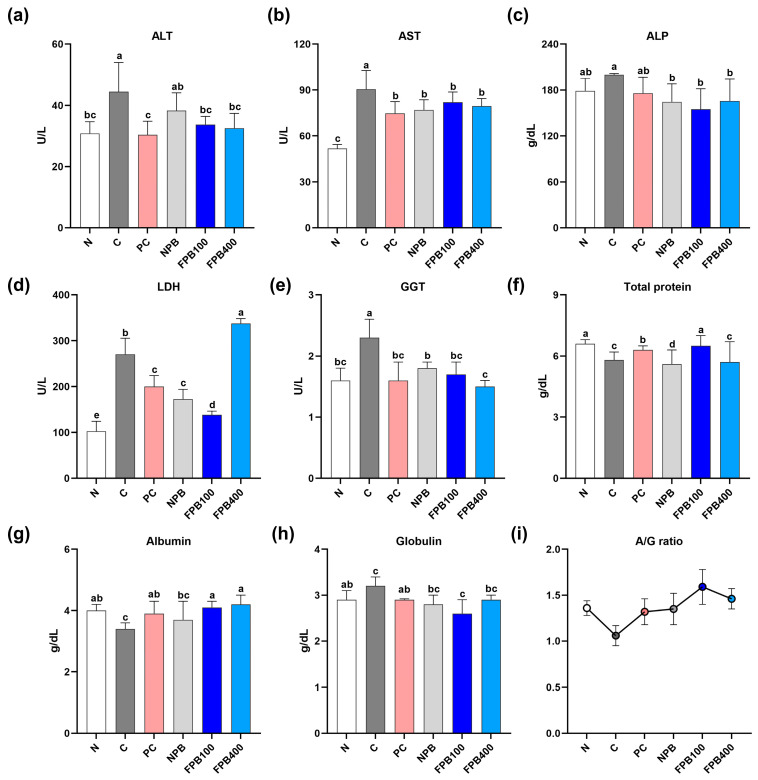
Effect of fermented *Protaetia brevitarsis* larvae (PbsLs) on the serum-based liver function indicators of alcohol-exposed rats: alanine aminotransferase (ALT; (**a**)), aspartate aminotransferase (AST; (**b**)), alkaline phosphatase (ALP; (**c**)), lactate dehydrogenase (LDH; (**d**)), gamma-glutamyl transferase (GGT; (**e**)), total protein (**f**), albumin (**g**), globulin (**h**), and the albumin/globulin (A/G) ratio (**i**). Values are presented as the mean ± standard error (*n* = 6). Means with the same letter are not significantly different (α = 0.05), as determined using one-way analyses of variance followed by Duncan’s new multiple-range tests. Treatments include N, normal; C, control (alcohol only); PC, positive control (silymarin); NPB, non-fermented PbsLs combined with *Bacillus subtilis* KACC 91157; and FPB100 and FPB400, 100 and 400 mg/kg/d, respectively, of PbsLs fermented by *B. subtilis* for 3 d.

Serum levels of aspartate aminotransferase (AST), alanine aminotransferase (ALT), and gamma-glutamyl transferase (GGT) were higher in all alcohol-administered groups than in the N group (Figure 3). The C group, to which only alcohol was administered, exhibited the highest levels, whereas the PC, NPB, and FPB groups showed relative reductions. Alkaline phosphatase (ALP) values were highest in the C group, and all other treatment groups exhibited decreased levels, with the FPB100 treatment producing the lowest. Regarding lactate dehydrogenase (LDH), all alcohol-administered groups showed higher levels than the N group, but the FPB400 group had the highest LDH level, with the C group having the second highest. The FPB100-group rats exhibited the lowest LDH levels. The total protein content results showed that all alcohol-administered groups, except for the FPB100 group, exhibited a decrease in total protein levels. In FPB100-group rats, total protein contents recovered to levels comparable to that of the N group. Alcohol consumption (C group) reduced albumin levels below that of normal rats (N group), and the PC, NPB, and FPB groups showed increased albumin levels compared to the C group, with the FPB groups showing the highest increases. On the other hand, the globulin content was highest in the C group and lowest in the FPB100 group, which was even lower than that of the N group. The albumin/globulin (A/G) ratio consistently showed elevated values in the FPB-treated groups.

### 3.8. Effect of FPBs on Components of the Alcohol Metabolism Pathway

**Figure 4:**
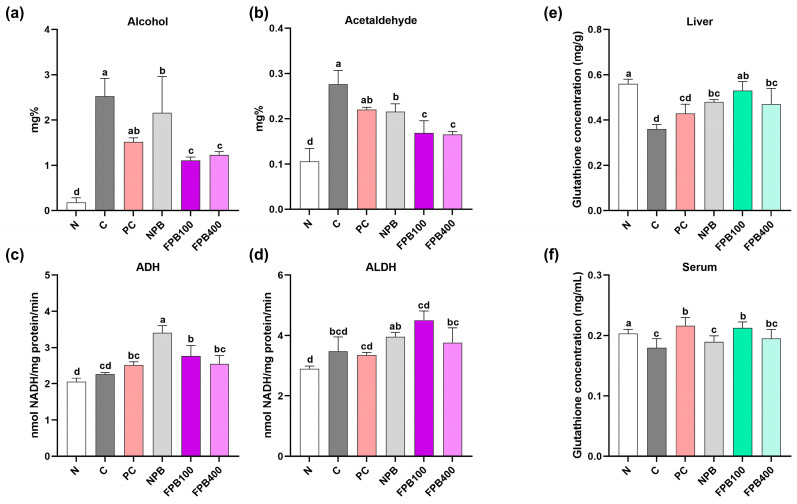
Effect of fermented *Protaetia brevitarsis* larvae (PbsLs) on the alcohol (**a**), acetaldehyde (**b**), alcohol dehydrogenase (ADH) (**c**), and aldehyde dehydrogenase (ALDH) (**d**) and glutathione concentrations in the liver (**e**) and serum (**f**) of alcohol-exposed rats. Values are presented as the mean ± standard error (*n* = 6). Means with the same letter are not significantly different (α = 0.05), as determined using one-way analyses of variance followed by Duncan’s new multiple-range tests. Treatments include N, normal; C, control (alcohol only); PC, positive control (silymarin); NPB, non-fermented PbsLs combined with *Bacillus subtilis* KACC 91157; and FPB100 and FPB400, 100 and 400 mg/kg/d, respectively, of PbsLs fermented by *B. subtilis* for 3 d.

The highest serum alcohol and acetaldehyde levels were observed in the C group, which can be attributed to alcohol consumption (Figure 4a–d). In the PC group, which received silymarin, alcohol and acetaldehyde levels were reduced, and the FPB groups consistently exhibited even lower alcohol and acetaldehyde levels, with the lowest alcohol level (1.11 mg/100 mL) observed in the FPB100 group. In this study, FPB treatment resulted in decreased serum alcohol and acetaldehyde levels. When ADH activity in liver tissue was measured, no noticeable difference was found between the N and C groups, but the NPB group showed the highest ADH content, and the FPB100 group had the second highest, though it was not significantly higher than that of the FPB400 group. Nevertheless, both the NPB- and FPB-treated groups exhibited high ADH contents. The N group had the lowest ALDH activity levels, while the FPB groups tended to show higher activity than the C group. However, as the FPB concentration increased to 400 mg/kg, ALDH levels greatly decreased.

### 3.9. Effect of FPBs on the Liver and Serum Glutathione Content

The C group exhibited the lowest glutathione levels in the liver due to alcohol intake. In contrast, the FPB treatments increased glutathione levels, with the FPB100 group yielding the highest. Although the FPB400 group showed slightly reduced liver glutathione levels, a significant difference was observed compared to the C group. Serum glutathione levels were lowest in the C group, while the FPB100 group showed a recovery of the glutathione content comparable to that of the PC groups, both of which produced levels comparable to that of the N group (Figure 4e,f).

### 3.10. Effect of FPBs on Hepatic and Serum Malondialdehyde (MDA) Levels

**Figure 5:**
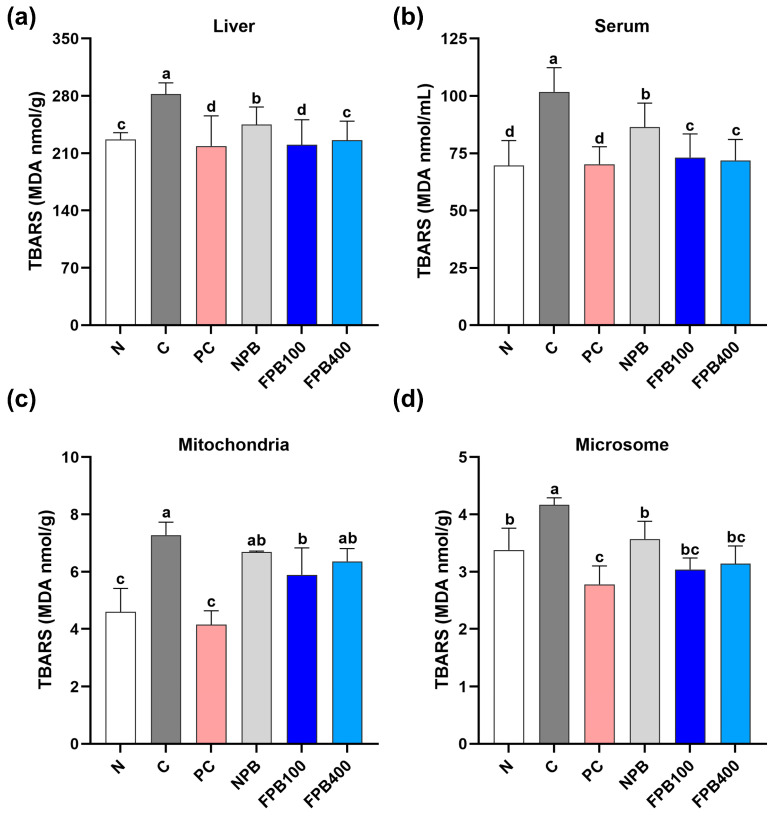
Effect of fermented *Protaetia brevitarsis* larvae (PbsLs) on the malondialdehyde (MDA) content in the liver (**a**), serum (**b**), mitochondria (**c**), and microsome (**d**) of alcohol-exposed rats. Values are presented as the mean ± standard error (*n* = 6). Means with the same letter are not significantly different (α = 0.05), as determined using one-way analyses of variance followed by Duncan’s new multiple-range tests. Treatments include N, normal; C, control (alcohol only); PC, positive control (silymarin); NPB, non-fermented PbsLs combined with *Bacillus subtilis* KACC 91157; and FPB100 and FPB400, 100 and 400 mg/kg/d, respectively, of PbsLs fermented by *B. subtilis* for 3 d.

The extent of cellular damage was determined by measuring the amount of MDA generated by peroxidation using thiobarbituric acid staining, and the results were quantified based on coloration (Figure 5). Liver, microsomes, mitochondria, and serum MDA levels were highest in the C group, which was administered alcohol only. In contrast, the PC group showed significantly reduced levels overall. All PbsL groups exhibited decreased MDA levels compared to the C group, with the FPBs-treated rats showing more pronounced reductions compared to the NPB-group rats. Although there was no significant difference in MDA levels between the FPB100 and FPB400 groups in serum, the FPB100 treatment reduced MDA levels more than the FPB400 treatment in the liver, microsomes, and mitochondria.

### 3.11. Effect of FPBs on Triglyceride Accumulation and Histopathological Alterations in Hepatic Tissue

**Figure 6:**
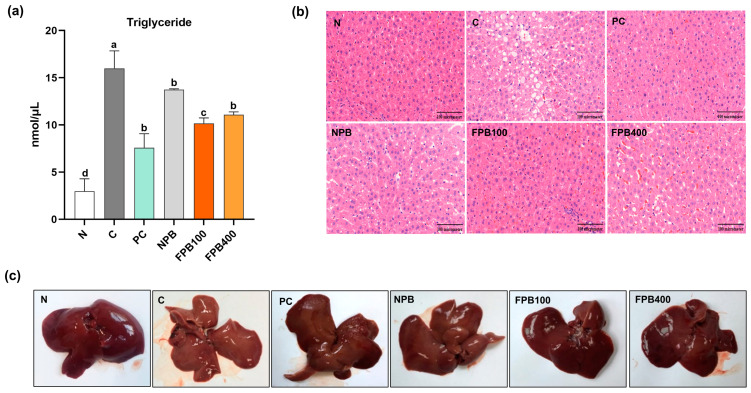
Effect of fermented *Protaetia brevitarsis* larvae (PbsLs) on the triglyceride content (**a**) of alcohol-exposed rats, along with histopathological images of their hepatic tissue (**b**) and images of their livers (**c**). Values presented in (**a**) are the mean ± standard error (*n* = 6). Means with the same letter are not significantly different (α = 0.05), as determined using one-way analyses of variance followed by Duncan’s new multiple-range tests. Treatments include N, normal; C, control (alcohol only); PC, positive control (silymarin); NPB, non-fermented PbsLs combined with *Bacillus subtilis* KACC 91157; and FPB100 and FPB400, 100 and 400 mg/kg/d, respectively, of PbsLs fermented by *B. subtilis* for 3 d.

The NPB treatment reduced triglyceride concentrations compared to that of the C group, and the PC group exhibited the lowest triglyceride concentration among the alcohol-administered treatments (Figure 6a). The PγG-enriched FPB groups reduced triglyceride contents to levels between these groups, with the FPB100 treatment yielded lower levels than the FPB400 treatment. In the N group, hepatic nuclei appeared round, and the structure of the liver lobules was regularly arranged (Figure 6b). In the C group, fat-droplet size increased, whereas in the PC group, it decreased, indicating that the development of fatty liver disease was suppressed. Although lipid droplet sizes significantly decreased in FPB-fed groups, reaching to 100 mg/kg in the FPB100 group, the higher FPB-concentration group displayed increased lipids. As shown in Figure 6c, liver appearance and firmness were observed using hepatic tissue from the rat model. The N group exhibited an overall reddish appearance without hepatic steatosis, and its tissue texture was notably firm. In contrast, the C group displayed prolific formation of hepatic steatosis, exhibiting a white appearance, and the tissue was easily disrupted by touch. The livers of the rats in the other experimental groups displayed an overall improved appearance compared with those of the C group, with the hepatic characteristics of the PC and FPB groups closely resembling those of the N group. These results provide insight into the ability of FPBs, enriched in PγG by the fermentation process, to improve alcoholic fatty liver disease when consumed.

## 4. Discussion

Alcohol is the primary cause of alcoholic fatty liver disease, contributing to the development of liver disorders in a significant portion of the population. Current research is focused on developing liver function enhancers and health-promoting foods aimed at preventing and alleviating this condition. Generally, single-component chemical compounds are prescribed to treat the symptoms of the disease. However, these can lead to a variety of side effects, including diarrhea, nausea, and vomiting. Consequently, natural products with complex components that exhibit effective treatment outcomes with fewer side effects are receiving increasing scientific attention. Although extensive research has focused on natural products for improving fatty liver, the potential of PbsLs to enhance the liver function lacked sufficient scientific evidence. Therefore, the present study applied a more innovative approach by incorporating microorganisms into PbsLs to investigate the effects of this natural product on improving alcoholic fatty liver disease through animal experiments. We selected the most suitable microorganisms for the fermentation of PbsLs by testing yeasts, lactic acid bacteria, molds, and filamentous fungi covering a wide taxonomic range to obtain fermented products and analyzed their antioxidant and anti-coagulant activities and bioactive substance contents to identify the optimal microorganism (Table 4 and Figure 1). The fermented product obtained from *B*. *subtilis* showed both the highest anti-coagulant activity and bioactive substance content, leading to the final selection of this species. Fermentation not only enhances the content of health-promoting antioxidant compounds, such as phenolics, but also has the advantage of improving their bioavailability [19]. According to the results of this study, phenolic and flavonoid contents were increased in all fermented groups after the inoculation of six different microorganisms into the insect powder. As previously mentioned, this enhancement is likely attributed to the synergistic effects of the fermentation process. A previous study on the phenolic compounds in *Protaetia brevitarsis* larvae reported the levels of 11 compounds, including vanillic acid and epicatechin [20]. Based on this, we anticipated that the fermentation process would lead to an overall increase in the content of these compounds. However, we acknowledge that a more thorough analysis and in-depth comparison of the actual contents are necessary for further clarification. The present study also determined the optimal fermentation duration for the maximum production of this polymeric substance, and a fermentation period of 3 days was selected. Based on the general microbial growth curve, which consists of the lag, exponential, stationary, and death phases, microbial growth is typically most rapid from the exponential phase to the early stationary phase. A previous study on the growth curve of *B*. *subtilis* isolated from soil reported that this transition occurs at approximately 3 days [21], supporting the selection of this fermentation duration in the present study. Additionally, from an industrial perspective, minimizing the overall processing time, including the fermentation period, while maintaining optimal efficacy, was a key consideration. Our experimental results validated this approach, as the FPB/D3 samples exhibited the highest levels of PγG, and cell-based assays demonstrated a significant reduction in adipogenesis at this time point. Lastly, since edible insects and larvae are rich in proteins and lipids, the prolonged fermentation process increases the risk of protein degradation and lipid oxidation. To mitigate these risks while maintaining fermentation effectiveness, the 3-day fermentation period was selected as the final fermentation duration in the present study.

Our findings demonstrate that fermentation effectively increased PbsLs’ ability to suppress alcohol-induced lipid accumulation in HepG2 cells (Table 5). *Bacillus* species typically produce PγG during growth, and the PγG content of FPBs was verified (Table 3). In a previous study, PγG administration to rats decreased their perirenal fat and abdominal fat. Furthermore, it decreased their hepatic cholesterol and triglyceride levels compared with the control [11]. This suggests that PγG administration may be closely related to suppressed hepatic lipogenesis. In another study, groups that fed on a high-fat diet containing PγG showed an increase in serum HDL cholesterol compared to the control group, while triglycerides decreased [22]. Additionally, PγG exhibits various biological activities, such as improving lipid metabolism and antiviral and antibacterial properties. Hypothesizing that high levels of PγG would influence hepatic lipid metabolism, Tamura et al. [23] showed that PγG can reduce liver lipids, indicating its potential as a component for improving fatty liver disease.

Based on the above research, we hypothesized that higher levels of PγG would mitigate alcoholic fatty liver disease. After determining optimal conditions, a 3-day-fermented product of PbsLs was obtained that contained approximately 38% more PγG compared to non-fermented PbsLs (Table 3). This product was then evaluated for its effectiveness in inhibiting lipid accumulation in liver cells using a cell-based assay. As anticipated, the 3-day-fermented PbsLs exhibited the lowest lipid production rate, which suggests that higher levels of PγG contribute to a synergistic inhibition of lipid accumulation. This is supported by previous research in which cholesterol and triglyceride levels in the liver were reduced by PγG consumption [11].

Given the evidence of a synergistic effect, we believed that not only the high content of PγG but also the presence of other beneficial components in FPBs would contribute to improving alcoholic fatty liver disease. To investigate this, an experiment was conducted using a rat model. In this study, to prevent acute alcohol toxicity, the alcohol concentration was gradually increased from 10% to 30% at weekly intervals. Male rats, aged 5 weeks, underwent a one-week adaptation period, followed by approximately three weeks of alcohol administration before being sacrificed for analysis. This study aimed to induce alcoholic fatty liver disease due to chronic alcohol intoxication while also evaluating its long-term effects. To ensure prolonged observation, infant-stage was excluded due to their rapid growth. Instead, we used 5-week-old male SD rats, as they are in the early maturation phase, making them more suitable for studying the long-term effects of alcohol consumption. When alcohol is consumed continuously, a common characteristic that emerges is a nutritional imbalance in the body leading to weight loss while simultaneously increasing liver weight due to fat accumulation. While the short-term consumption of large amounts of alcohol can suppress fatty acid oxidation, resulting in weight gain, chronic consumption can lead to weight loss due to increased fatty acid oxidation and energy expenditure [24]. As absolute liver weight alone cannot accurately assess liver enlargement, relative liver weight is calculated as the liver weight divided by the body weight, expressed as a percentage. Additionally, when comparing relative liver weights among experimental groups, variations in body weight among individuals can add to the variance of the data, and relative liver weight accounts for this. A study evaluating the hepatoprotective activity of an ethanol extract of *Pterocarpus marsupium* Roxb. leaves against paracetamol-induced liver damage also used the relative liver weight to body weight to analyze the extent of liver damage [25]. When 100 mg/kg of FPBs were administered concurrently with alcohol, it not only alleviated the weight loss commonly observed with chronic alcohol consumption but also suppressed the formation of a fatty liver, resulting in a lower relative liver weight compared to those of rats to which only alcohol was administered. In contrast, a high concentration of FPBs reduced its alleviatory effect on weight loss, yet the relative liver weights of rats receiving 400 mg/kg were similar to those of the silymarin-administered rats, indicating an ability to inhibit fatty liver formation (Table 6).

Alcohol has many side effects, such as obesity, high blood pressure, diabetes, and fatty liver disease. Additionally, alcohol is a significant risk factor for cardiovascular disease, with various complications like high cholesterol, hypertension, and hyperlipidemia [26]. Low HDL cholesterol levels can cause cardiovascular diseases, such as hypertension and arteriosclerosis, while increased LDL cholesterol and triglyceride levels potentially lead to hyperlipidemia [27]. Qiao et al. [28] elucidated the mechanism by which alcohol and PbsL consumption affect cardiovascular indicators. Alcohol consumption potentially increases total and LDL cholesterol levels, leading to heart disease. In this study, the results indicated that the total cholesterol and LDL cholesterol levels in the FPB400 group were higher than those in the FPB100 group. This observation suggests that when the natural compound is used in excess, it may exhibit an opposite trend compared to the optimal dosage. However, the arterial stiffness index decreased by approximately 36% in the PbsL-fed animals. We found an even greater reduction in the atherogenic index, an indicator of arteriosclerosis, in the rats to which FPBs were administered in this study (Figure 2a–c). This finding is consistent with that of another study identifying PbsLs’ role in decreasing triglyceride levels [29].

The FPB-fed groups yielded decreased serum triglyceride levels, with the lowest total lipid, free fatty acid, and triglyceride levels detected in the FPB100-group rats (Figure 2d–f). Triglycerides account for a vast proportion of the fat cells in the body. When the body’s energy source is insufficient, triglycerides composed of neutral fat decompose into glycerol and free fatty acids. Consequently, the serum-free fatty acid concentration increases [30]. Via β-oxidation, the free fatty acids generate energy, which is subsequently used by the body [31]. In the present study, alcohol was administered to rats to artificially induce fatty liver disease, and the triglyceride and free fatty acid concentrations rapidly increased. In contrast, when FPBs were concurrently administered, enhanced PγG contents and decreased serum triglyceride and free fatty acid concentrations were observed, and the lowest concentrations were observed in the 100 mg/kg FPB-fed rats. Edible insects and larvae generally contain high levels of proteins and lipids. Therefore, when absorbed at concentrations above the optimal level, rather than reducing lipid contents such as triglycerides and free fatty acids, they tend to show opposing effects. Furthermore, the diverse effects of various natural products have been extensively studied [32], and it has been reported that some natural products at high concentrations may exhibit toxicity or adverse effects in the body. This suggests that the 100 mg/kg FPB treatment was more effective than the higher FPB concentrations. Furthermore, this observation raises the possibility that for insect powders as well as other natural products, optimal effects may be achieved at appropriate doses, rather than higher concentrations. Thus, the FPB-fed rats had a greater ability to decompose triglycerides than the non-FPB-fed group. Another study reported significant decreases in the serum triglyceride and free fatty acid concentrations in PγG-fed rats [22]. These findings corroborate ours, which demonstrated improved serum lipid metabolism in rats fed a PγG-enriched FPB diet.

Albumin typically constitutes approximately 50% of the proteins in plasma and plays a crucial role in blood pressure regulation and osmotic pressure control [33]. In addition, it is an extracellular protein that tends to decrease in cases of severe liver disease, thus serving as a crucial indicator of liver dysfunction [34]. Additionally, in cases of chronic liver disease, albumin synthesis is known to decrease, highlighting the need for research aimed at finding ways to increase its levels [35]. The PbsL-administered experimental groups (NPB and FPB) showed an overall increase in albumin levels (Figure 3g), which is consistent with previous research indicating an increase in albumin content in high-fat diet mice fed hydrolysates derived from PbsLs [36]. This further suggests that the fermentation method employed in this study was effective.

Increased serum ALT, AST, ALP, GGT, and LDH activities are also clinical indicators of liver damage. Furthermore, serum ALT and AST activities are specifically tied to liver cell necrosis [37]. In a study that reported the effect of high molecular weight PγG on hypertriglyceridemia in fructose-fed rats, high PγG concentrations returned AST and ALT levels to close to those of the N group [11]. Alkaline phosphatase not only exists in the villi and epithelial tissue of the hepatic bile duct but is also involved in promoting skeletal calcification [38]. An elevated serum ALP level is a major feature of alcoholic liver disease and bone disease [39]. Serum GGT activity is also elevated in fatty liver disease, which predisposes individuals to alcohol-related diseases [40]. Furthermore, GGT activity exceeds that of other enzymes when the liver or gallbladder is diseased [41]. Lactate dehydrogenase activity is linked to glycolysis, which increases acute hepatitis and liver cancer risk [42]. Our results verify that both PbsLs and FPBs alleviated the elevated liver function enzyme levels induced by alcohol (Figure 3a–e).

The liver contains enzymes, such as ADH and ALDH, that are crucial for alcohol metabolism. When alcohol is oxidized by ADH, acetaldehyde is produced [43]. A hangover may result from the substantial accumulation of acetaldehyde in the liver and blood circulation. Aldehyde dehydrogenase plays a significant role in the liver by converting acetaldehyde into acetic acid, which is largely released into the blood circulation, while a portion is excreted via urine or as carbon dioxide [44]. Overall, alcohol is eliminated throughout this procedure. During alcohol oxidation, NADH and acetaldehyde are generated. NADH, which is the reduced form of NAD+ produced by the alcohol oxidation mechanism, induces lipogenesis in liver cells [45]. The accumulation of acetaldehyde, which is an intermediate product of alcohol decomposition, is toxic to the body [46]. Therefore, continuous alcohol intake increases body acetaldehyde and NADH levels and promotes liver cell damage [28]. In this study, alcohol, acetaldehyde, ADH, and ALDH levels were identified as indicators of the rat’s ability to decompose alcohol (Figure 4a–d). Compared with non-fermented powder, silkworm powder fermented using *B. subtilis* exhibited higher ADH and ALDH activity [47]. The present study used another type of insect larvae, PbsL, combined with *B. subtilis* for fermentation, and similar results were observed. These findings suggest a synergistic effect between insect larvae and microorganisms in mitigating alcohol-induced liver damage.

Glutathione is known to exhibit numerous biological activities, including antioxidant, anti-inflammatory, and immune-enhancing properties, which serve crucial roles in the body [48]. Notably, it removes toxins within the liver, helping to maintain and protect the liver function. The glutathione’s response to the oxidative stress induced by alcohol intake is considered a cellular protective mechanism. In a study where mealworm larvae fermented using yeast were administered to alcohol-fed rats, an enhancement of the antioxidant defense system, including glutathione, was observed within the liver [49]. In the present study, FPBs similarly increased glutathione levels (Figure 4e,f). Mitochondria in the liver play a crucial role in breaking down substances such as carbohydrates, proteins, and lipids to generate energy for cellular supply [50]. To study liver metabolism, various cellular fractions, such as microsomes and cytosol, are typically examined, with microsomes, which contribute to the synthesis of proteins, lipids, and other cellular components in the liver, being widely used. The functions of mitochondria and microsomes were examined in the present study (Figure 5a–d). A recent study by Sastre et al. found increased malondialdehyde levels in mitochondria from rats on an ethanol diet for 6 weeks, indicating that chronic ethanol consumption enhances oxidative stress products in mitochondria [51]. Similarly, in the present study, the MDA levels were highest in the C group, while the FPB-treated group showed significantly lower levels. We estimated the extent of alcohol-induced cellular damage by measuring the amount of MDA generated through peroxidation using thiobarbituric acid staining, with results quantified based on coloration. When the liver is damaged due to factors such as alcohol consumption, functional abnormalities in cellular organelles occur. In the present study, it was confirmed that the administration of FPBs inhibited the production of lipid peroxides in liver cell fractions. Alcoholic fatty liver disease refers to the enlargement of the liver in response to the accumulation of more than 5% triglycerides [52]. While various factors contribute to fatty liver disease, alcohol intake is the most common. According to a study investigating the effects of PγG on the lipid metabolism of rats fed a high-fat diet, PγG-fed rats experienced a decrease in hepatic triglyceride levels [10]. Ahn et al. (2019) revealed reduced hepatic triglyceride levels owing to PbsL administration in rats. The FPB-fed rats in this study displayed greater abilities to decompose triglycerides than the PbsL-fed rats (Figure 6a). This demonstrates the potential of PγG-enriched FPB consumption to improve alcoholic fatty liver disease. Chronic alcohol consumption leads to an imbalance in oxygen and nutrient levels within the liver [53], which contributes to hepatic lipid accumulation. Changes in the organization of liver cells during the initial stages of ALD can be discerned from alterations in the accumulation of fat droplets within these cells. Small fat droplets have been reported to expand into larger ones, thereby inducing fatty liver disease. This process can subsequently result in hepatitis and progress to the final stage of cirrhosis [54]. In this study, liver tissue stained with H&E showed that FPB consumption resulted in a regular arrangement of liver lobules and a reduction in fat droplet size, despite a concurrent high consumption of alcohol (Figure 6b). Moreover, visual inspection of actual liver samples revealed less hepatic steatosis in the rats administered FPBs, with the liver appearing red and firm (Figure 6c). These findings validate FPBs’ potential to improve alcoholic fatty liver disease.

Since both alcohol-related and non-alcohol-related factors contribute to fatty liver development, this study evaluated the efficacy of FPBs in mitigating alcoholic fatty liver while building on previous findings regarding its effects on the non-alcoholic fatty liver. To achieve this, various *in vitro* experiments were first conducted to compare the functional properties of different samples, and the most suitable sample was subsequently selected for application in an animal model, with experiments designed accordingly. Rather than focusing on specific molecular mechanisms, this study assessed physiological changes using both *in vitro* and *in vivo* approaches to better reflect pathological conditions. The consistent effects of FPBs across both models further support its therapeutic potential. Numerous studies have reported the effects of various interventions on fatty liver improvement, and research on the mechanisms by which TLR-4/TGF-β1 modulates alcohol-induced hepatotoxicity has also been conducted [55]. Similarly, studies investigating the hepatoprotective effects of various factors beyond alcohol-induced toxicity are actively ongoing. In most cases, liver dysfunction improvement is analyzed in relation to mechanisms such as lipid metabolism, antioxidant activity, or anti-inflammatory responses. However, this study aims to discuss several possible considerations.

First, based on the results of PγG content measurement in the final product selected for this study and previous research on this compound, we propose its potential role in improving fatty liver disease. While the comparison study analyzed or represented the amino acid content of FPBs, this study not only examined the differences before and after fermentation but also focused on PγG, a secondary metabolite predominantly produced by *B*. *subtilis*, identifying an increase in the glutamic acid content. This increase in glutamic acid is directly linked to the improvement of the fatty liver. Additionally, we referred to studies on the lipid-improving effects of PγG, further supporting its relevance to this study. Second, although mechanisms such as antioxidant, anti-inflammatory, and lipid-improving activities are commonly identified as contributors to fatty liver amelioration, as mentioned in the discussion section of this manuscript, the improvement of the fatty liver in this study was assessed based on observable changes in animals. The body is a highly complex system influenced by various enzymes; thus, realistic outcomes such as microscopic or anatomical observations are equally important. For this reason, we compared histopathological images and real liver tissue images in our study. Third, this study not only analyzed serum but also examined changes in liver tissue. More specifically, by fractionating the liver into mitochondria and microsomes, a clearer interpretation of lipid metabolism changes was achieved, including the peroxidized lipid content, triglyceride levels, and microscopic observations. Finally, in the comparative study, chronic alcohol toxicity in the mouse model was induced by administering alcohol at a constant concentration for 8 weeks. However, in this study, despite providing alcohol to the rat model for only approximately 3 weeks with a gradual increase in concentration, the clear induction of the fatty liver was observed.

The publication of the aforementioned study provides a valuable reference point for distinguishing the significance of our study. The reasons are as follows: First, this study demonstrates that the effects of FPBs can be observed even at lower concentrations, while higher concentrations exhibit opposing results in lipid metabolism improvement. This allows for a more detailed assessment of FPBs’ effects across various sample concentrations. Second, in addition to elucidating the mechanisms underlying lipid metabolism improvement and antioxidant activity in chronic alcohol-induced conditions, this study provides a more precise analysis of fatty liver improvement through histological and anatomical observations of hepatic morphology. Lastly, by confirming alcohol-induced liver damage in two different animal models (rats and mice), to which alcohol was continuously administered for three and eight weeks, respectively, and verifying the effects of FPBs, this study serves as a valuable foundation for future in-depth research. Future studies will greatly contribute to a clearer understanding of the efficacy of PγG and FPBs by analyzing changes in the bioactive compound content and investigating lipid metabolism mechanisms, thereby elucidating their role in improving fatty liver disease.

## 5. Conclusions

This study demonstrated that externally administered FPBs effectively improves alcoholic fatty liver disease by reducing hepatic fat accumulation and enhancing the liver function. Furthermore, it investigated the therapeutic effects of FPBs on alcoholic fatty liver disease induced by chronic alcohol consumption through comprehensive analysis of serum and hepatic biomarkers, as well as histological and anatomical examinations. Additionally, this study confirmed that the content of PγG produced by *Bacillus subtilis* increased when combined with PbsLs. Previous research has suggested that PγG contributes to the amelioration of fatty liver disease, further supporting the potential benefits of FPBs. Based on *in vitro* findings, this study selected and analyzed samples for use in an animal model. To induce chronic alcohol toxicity, 5-week-old male Sprague–Dawley (SD) rats were subjected to a gradual increase in alcohol concentration, reaching 30% over approximately three weeks. This approach was designed to align with previous research on the beneficial effects of FPBs in non-alcoholic fatty liver disease, thereby enhancing the reliability of this study by employing a comparable animal model. These findings highlight the potential of FPBs as a functional food ingredient with ameliorative effects against alcoholic hepatic steatosis (AHS) and suggest that FPB treatment is effective in improving alcoholic fatty liver disease. Thus, FPBs may serve as a promising therapeutic approach for mitigating alcohol-induced liver damage.

## Figures and Tables

**Table 1:** Experimental diet composition.

Ingredients (%)									
Casein	Corn Starch	Corn Oil	Cellulose	Mineral ^(1)^	Vitamin ^(2)^	L-Methionine	Sucrose	Choline	Total
20.0	15.0	10.0	5.0	4.0	1.0	0.3	44.5	0.2	100

^(1)^ AIN 93 M-MX mineral mix, MP biomedicals, Illkirch, France; ^(2)^ AIN 93 VX vitamin mix, MP biomedicals.

**Table 2:** Experimental treatments for the AHS rat model.

Group	Composition
N	normal diet
C	normal diet + Alcohol
PC	normal diet + Alcohol + Silymarin
NPB	normal diet + Alcohol + NPB 400 mg/kg b.w./day
FPB100	normal diet + Alcohol + FPB 100 mg/kg b.w./day
FPB400	normal diet + Alcohol + FPB 400 mg/kg b.w./day

Alcohol was 20% (*w*/*v*) ethyl alcohol administered at 5 g/kg. Abbreviations: NPB, non-fermented *P. brevitarsis* combined with *B. subtilis* KACC 91157; FPB, *P. brevitarsis* larvae fermented by *B. subtilis* for 3 d; b.w., body weight.

**Table 3:** Poly-γ-glutamic acid (PγG) contents of *Protaetia brevitarsis* larvae (PbsL) powder at different fermentation durations.

PγG Content				
Group	NPB	FPB/D1	FPB/D2	FPB/D3
mg/g	47.17 ± 0.02 ^c^	50.31 ± 0.01 ^b^	64.74 ± 0.01 ^a^	65.50 ± 0.01 ^a^

The values presented are the mean ± standard error (*n* = 3). Values with the same letter are not significantly different (α = 0.05), as determined using a one-way analysis of variance followed by Duncan’s new multiple-range test. Treatments: NPB, non-fermented PbsLs; FPB/D1, PbsLs fermented by *Bacillus subtilis* for 1 d; FPB/D2, PbsLs fermented by *B. subtilis* for 2 d; FPB/D3, PbsLs fermented by *B. subtilis* for 3 d.

**Table 4:** Antioxidant and antithrombotic activities of aqueous fermented *Protaetia brevitarsis* larvae (PbsL) extracts according to microbial species used in fermentation.

Group	DPPH Radical-Scavenging Activity	Antithrombotic Activity
	(%)	(Unit)
BHT	87.90 ± 1.36 ^a^	-
N	14.05 ± 0.52 ^b^	2.88 ± 0.17 ^a^
F3	55.69 ± 1.94 ^ce^	39.20 ± 0.55 ^b^
F5	56.98 ± 0.25 ^c^	39.20 ± 0.60 ^b^
Ba9	57.81 ± 1.49 ^c^	42.00 ± 0.58 ^b^
Ak	46.33 ± 0.48 ^d^	33.80 ± 0.44 ^c^
Sc	53.89 ± 2.81 ^a^	33.80 ± 0.62 ^d^
Bs	50.58 ± 2.61 ^c^	64.80 ± 0.74 ^e^

The values presented are the mean ± standard error (*n* = 3). Values in a given column with the same letter are not significantly different (α = 0.05), as determined using one-way analyses of variance followed by Duncan’s new multiple-range tests. Abbreviations include BHT, butylated hydroxytoluene; N, non-fermented PbsLs; F3, PbsLs fermented by *Lactobacillus plantarum* JBMI F3 for 3 d; F5, PbsLs fermented by *L. plantarum* JBMI F5 for 3 d; Ba9, PbsLs fermented by *L. gaseri* Ba9 F3 for 3 d; Ak, PbsLs fermented by *Aspergillus kawachii* KCCM 32819 for 3 d; Sc, PbsLs fermented *by Saccharomyces cerevisiae* KACC 93023 for 3 d; Bs, PbsLs fermented by *Bacillus subtilis* KACC 91157 for 3 d.

**Table 5:** Inhibitory effect of fermented *Protaetia brevitarsis* larvae (PbsLs) on hepatic lipid accumulation in HepG2 cells stained with Oil Red O.

Oil Red O Absorbance						
Group	N	C	NPB	FPB/D1	FPB/D2	FPB/D3
% of control	100.00 ± 6.46 ^d^	305.50 ± 11.68 ^a^	257.80 ± 6.49 ^b^	235.78 ± 11.68 ^b^	234.86 ± 7.78 ^b^	179.82 ± 28.54 ^c^

The values presented are the mean ± standard error (*n* = 3). Values with the same letter are not significantly different (α = 0.05), as determined using a one-way analysis of variance followed by Duncan’s new multiple-range test. Abbreviations include N, normal; C, control; NPB, non-fermented PbsLs combined with *Bacillus subtilis*, FPB/D1, PbsLs fermented by *B. subtilis* for 1 d; FPB/D2, PbsLs fermented by *B. subtilis* for 2 d; FPB/D3, PbsLs fermented by *B. subtilis* for 3 d.

**Table 6:** Body weights, relative liver weights, and water consumption in the experimental rats.

Group	Body Weight (g)	Relative Liver Weight (% of Terminal Body Weight)	Water Consumption (mL/day)
N	166.33 ± 7.37 ^a^	3.33 ± 0.24 ^bc^	35.88 ± 6.77 ^a^
C	124.33 ± 10.41 ^e^	3.76 ± 0.41 ^a^	25.43 ± 3.41 ^b^
PC	156.33 ± 10.79 ^c^	3.33 ± 0.29 ^bc^	23.75 ± 2.36 ^b^
NPB	144.67 ± 14.19 ^d^	3.51 ± 0.23 ^ab^	26.83 ± 4.62 ^b^
FPB100	161.67 ± 27.68 ^b^	3.08 ± 0.26 ^d^	29.50 ± 7.92 ^b^
FPN400	125.67 ± 13.05 ^e^	3.33 ± 0.21 ^bc^	22.50 ± 2.95 ^b^

The values are presented as the mean ± standard error (*n* = 6). Values within a given column with the same letter are not significantly different (α = 0.05), as determined using one-way analyses of variance followed by Duncan’s new multiple-range tests. Abbreviations include N, normal; C, control (alcohol only); PC, positive control (silymarin); NPB, non-fermented, *Protaetia brevitarsis* larvae (PbsLs); FPB100, 100 mg/kg PbsLs fermented by *B. subtilis* for 3 d; FPB400, 400 mg/kg PbsLs fermented by *B. subtilis* for 3 d.

## Data Availability

The original contributions presented in this study are included in the article/Supplementary Material. Further inquiries can be directed to the corresponding author.

## Supplementary Materials

The following supporting information can be downloaded at: https://www.mdpi.com/article/10.3390/foods14050861/s1, Table S1: Protein-bound amino acids composition (mg/100 g) in PbsL and FPB; Table S2: Free amino acids composition (mg/100 g) in PbsL and FPB.
