# Supplementary material for: Amelioration of Alcoholic Hepatic Steatosis in a Rat Model via Consumption of Poly-γ-Glutamic Acid-Enriched Fermented Protaetia brevitarsis Larvae Using Bacillus subtilis
Source: Foods. 2025 Mar 3;14(5):861. doi: 10.3390/foods14050861 (PMC11899319; doi:10.3390/foods14050861)
Supplement: Supplementary file 1 [file foods-14-00861-s001.zip › foods-3466321-supplementary.pdf]

Table S1. Protein-bound amino acids composition (mg/100 g) in PbsL and FPB.

Protein-bound amino acids mg/100 g	Group	
	PbsL	FPB
Aspartic acid	2701.834	2709.899
Threonine	1551.689	1508.276
Serine	2115.991	2151.848
Glutamic acid	4830.888	5436.014
Proline	5644.353	5785.922
Glycine	2413.204	2252.994
Alanine	1728.306	1550.864
Cystine	0.000	0.000
Valine	1901.246	1937.473
Methionine	535.915	567.989
Isoleucine	1464.835	1472.525
Leucine	2078.272	2101.339
Tyrosine	3645.194	4101.709
Phenylalanine	1560.250	1611.296
Histidine	3365.569	3387.495
Lysine	2040.868	2151.157
Arginine	1642.062	1465.410
Total	39220.476	40192.210

Table S2. Free amino acids composition (mg/100 g) in PbsL and FPB.

Free amino acid mg/100 g	Group	
	PbsL	FPB
Phosphoserine	0.000	0.000
Taurine	0.000	0.000
Phosphoethanolamine	0.000	0.000
Urea	0.000	0.840
Aspartic acid	0.000	0.106
Hydroxyproline	0.000	0.000
Threonine	3.989	7.683
serine	0.000	0.512
Asparagine	0.000	0.189
Glutamic acid	0.940	1.386
Sarcocine	0.000	0.000
α-aminoadipic acid	0.000	0.208
Proline	82.897	186.982
Glycine	10.540	17.246
Alanine	8.252	19.500
Citrulline	0.000	0.000
α-aminobutyric acid	0.000	0.825
Valine	7.761	45.843
Cystine	0.000	0.000
Methionine	0.000	3.840
Isoleucine	3.391	26.803
Leucine	0.159	30.909
Tyrosine	1.093	9.233
phenylalanine	1.291	11.292
β-alanine	0.000	0.428
β-aminoisobutyric acid	0.000	0.000
γ-amino-n-butyric acid	0.000	0.767
Histidine	0.923	3.063
3-methylhistidine	0.000	0.000
1-methylhistidine	0.000	0.000
Carnosine	0.000	6.780
Anserin	0.000	0.000
Tryptopan	0.000	0.000
Hydroxylysine	0.000	0.000
Ornitine	0.000	0.000
Lysine	0.000	0.000
Arginine	0.000	0.000
Total	121.236	374.435
